# Supplementary figures and images for: A genetic screen identifies a protective type III interferon response to Cryptosporidium that requires TLR3 dependent recognition
Source: PLoS Pathog. 2022 May 18;18(5):e1010003. doi: 10.1371/journal.ppat.1010003 (PMC9154123; doi:10.1371/journal.ppat.1010003)

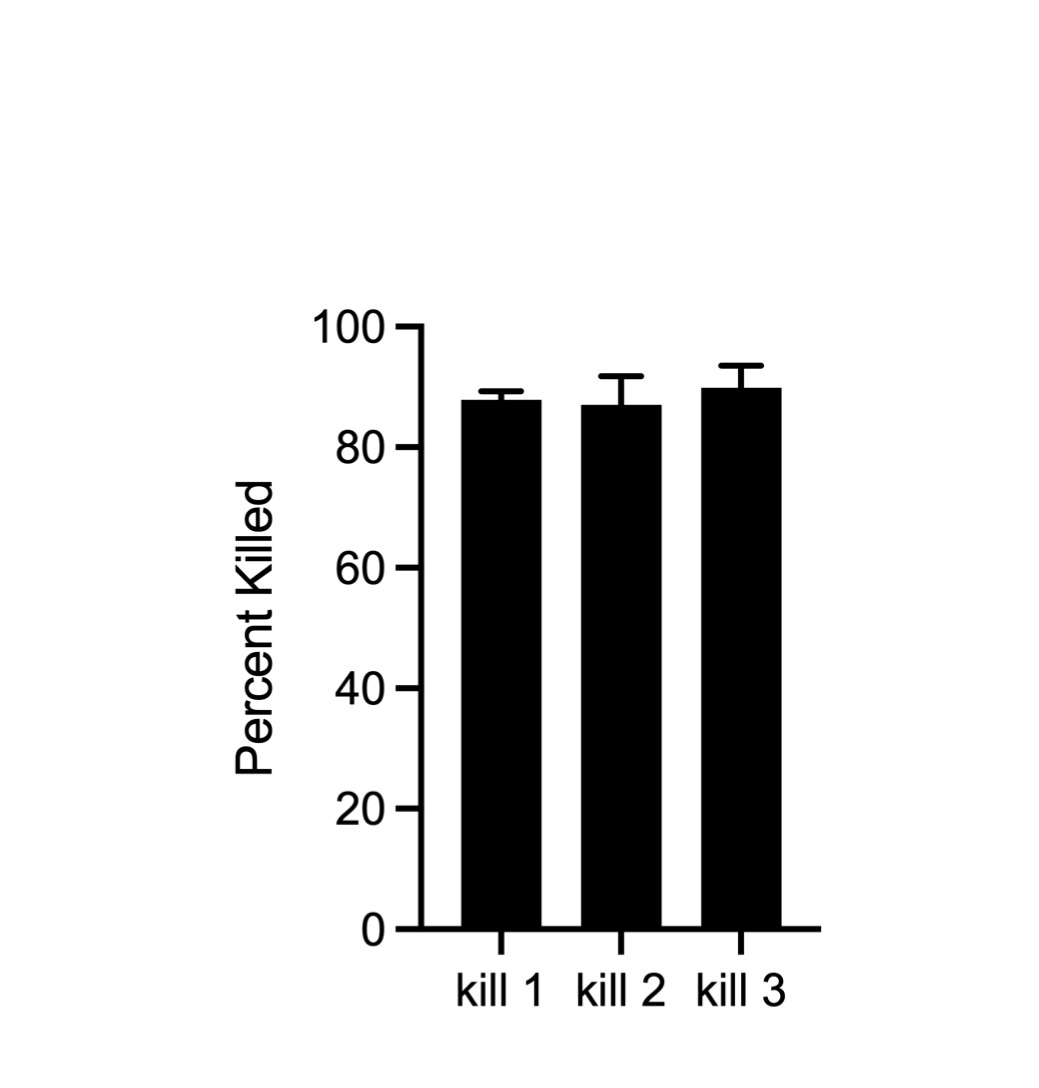

Supplement: S1 Fig — Cas9 expressing HCT-8 cells were infected successively with a 90% kill dose (MOI = 3) but in contrast to the screen this was done in the absence of the sgRNA library. Host cell viability was assessed by Trypan Blue exclusion and no change in susceptibility was observed under these conditions. (TIF) [file ppat.1010003.s001.tif]

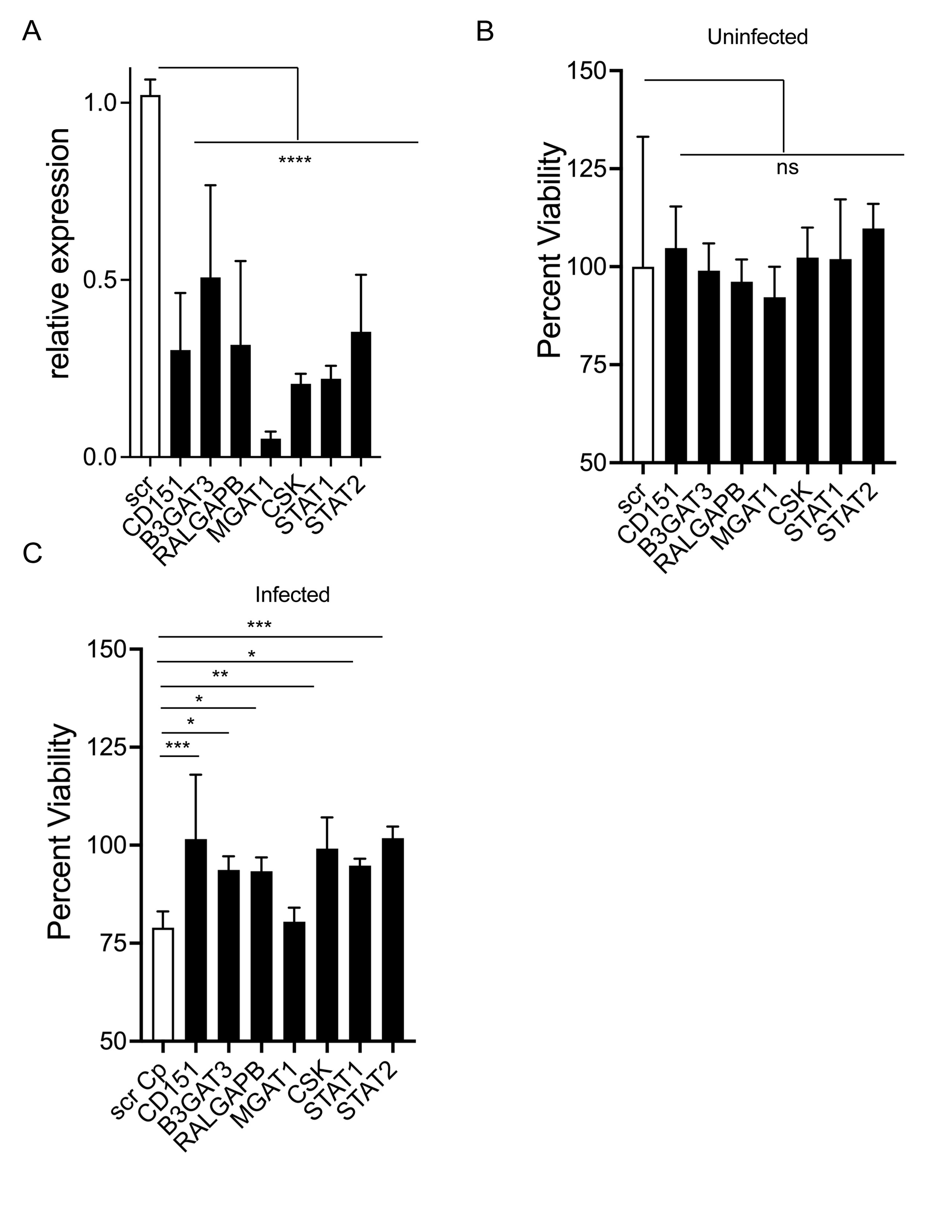

Supplement: S2 Fig — We used siRNA treatment to knockdown transcripts of genes identified in our screen and assessed host cell viability following infection. (A) Relative expression of genes targeted for knockdown normalized to the scrambled (scr) siRNA control. n = 2. (B) Knockdown of top candidates does not affect host cell viability in the absence of infection. MTT assay normalized to uninfected scrambled (scr) siRNA control. n = 2. (C) Knockdown of candidates leads to an increase in host cell viability during C. parvum infection. MTT assay normalized to uninfected scrambled (scr) siRNA control. n = 2. (TIF) [file ppat.1010003.s002.tif]

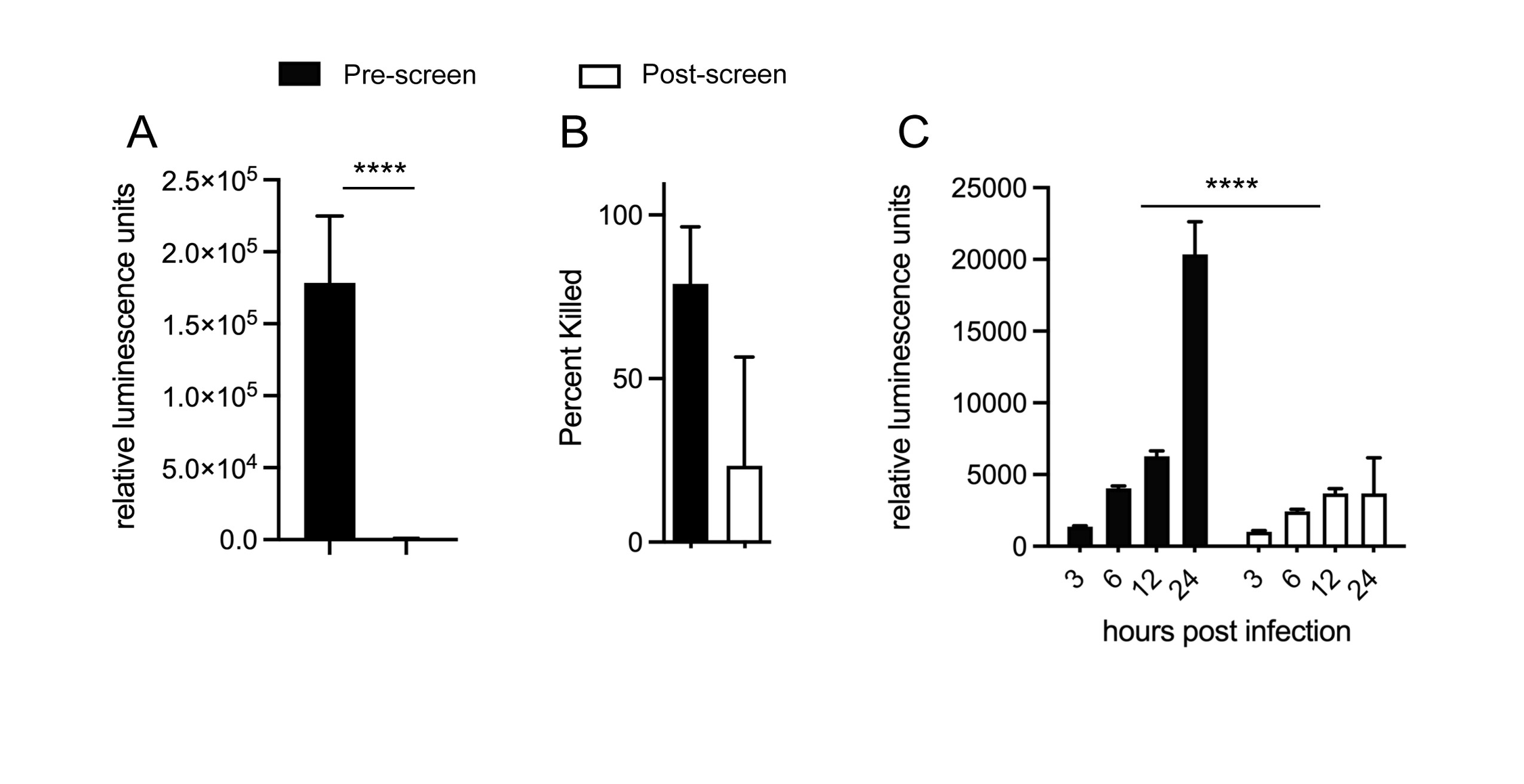

Supplement: S3 Fig — The population of Cas9-Clone K expressing cells transduced with the library prior to the screen were compared to the population following the last round of selection. (A) The pre- and post-screen populations were infected with Nanoluciferase expressing parasites for 48 hours. Parasite growth was measured by Nanoluciferase assay. Standard t-test p < 0.001 **** n = 3. (B) Cells were infected with C. parvum for 72 hours. Host cell viability was assessed by Trypan Blue exclusion. n = 3 Standard t-test. (C) The pre- and post-screen populations were infected with Nanoluciferase expressing parasites for the indicated time points. Parasite growth was measured by Nanoluciferase assay. Two-way ANOVA p < 0.001 **** n = 3. (TIF) [file ppat.1010003.s003.tif]

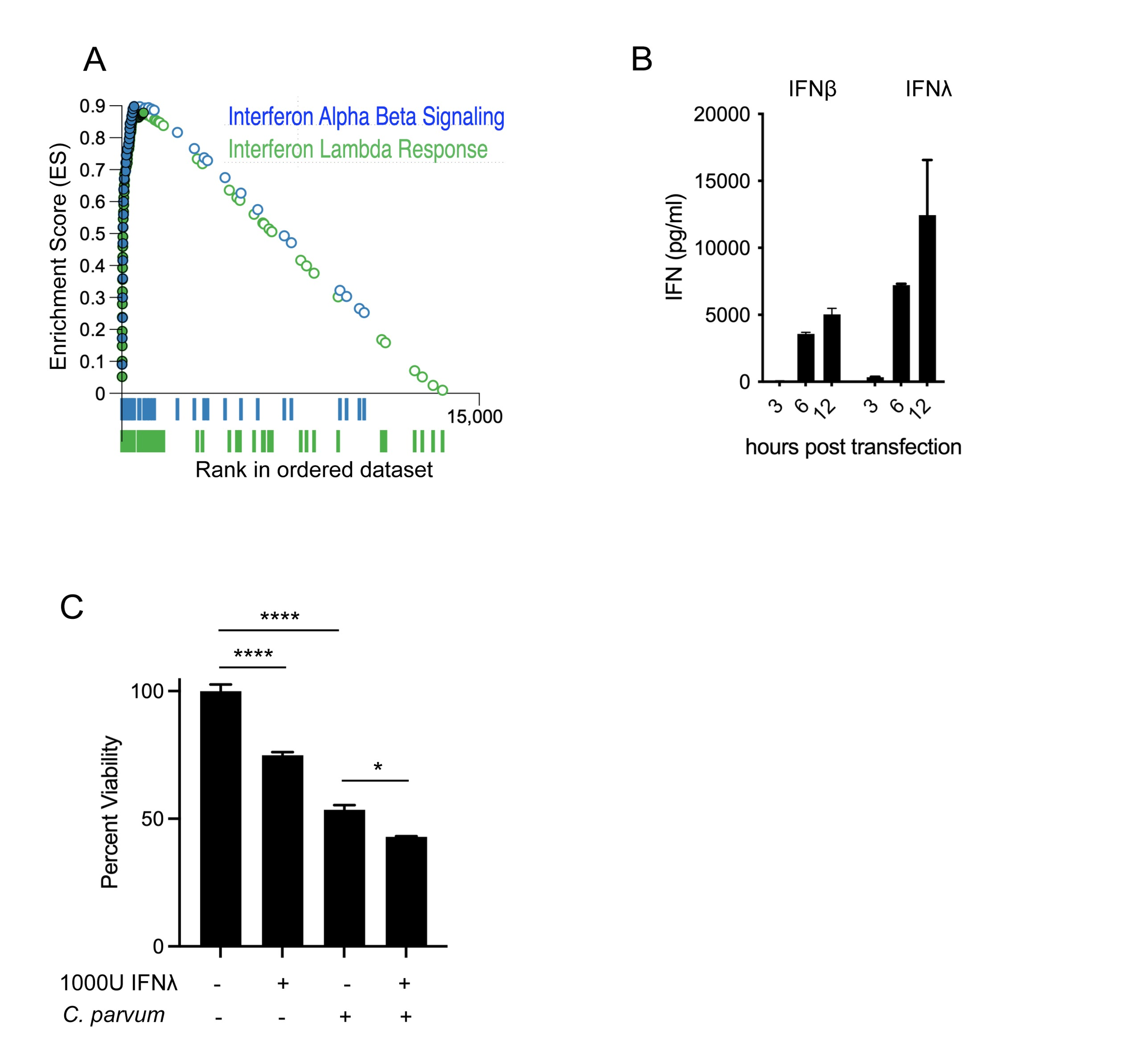

Supplement: S4 Fig — (A) GSEA plot showing Interferon Alpha Beta Signaling and Interferon Lambda Response signatures identified at 48 hours post infection. Closed circles represent genes that make up the core enrichment of the signature. Note that many of the genes overlap. Alpha Beta: Net enrichment score = 2.9, p-value <0.0001, Lambda: Net enrichment score = 3.04, p value <0.0001. (B) Protein levels of IFNβ and IFNλ following lipofection with 10μg/mL Poly(I:C) as measured by ELISA. Note the maximal production of IFNβ is 27-fold higher than that observed during C. parvum infection. (C) MTT assay normalized to untreated, uninfected control. HCT-8 were infected for 48 hours following 16 hours treatment with IFN at the indicated doses. One-way ANOVA with Dunnett’s multiple comparisons test * p <0.05 **** p <0.0001. (TIF) [file ppat.1010003.s004.tif]

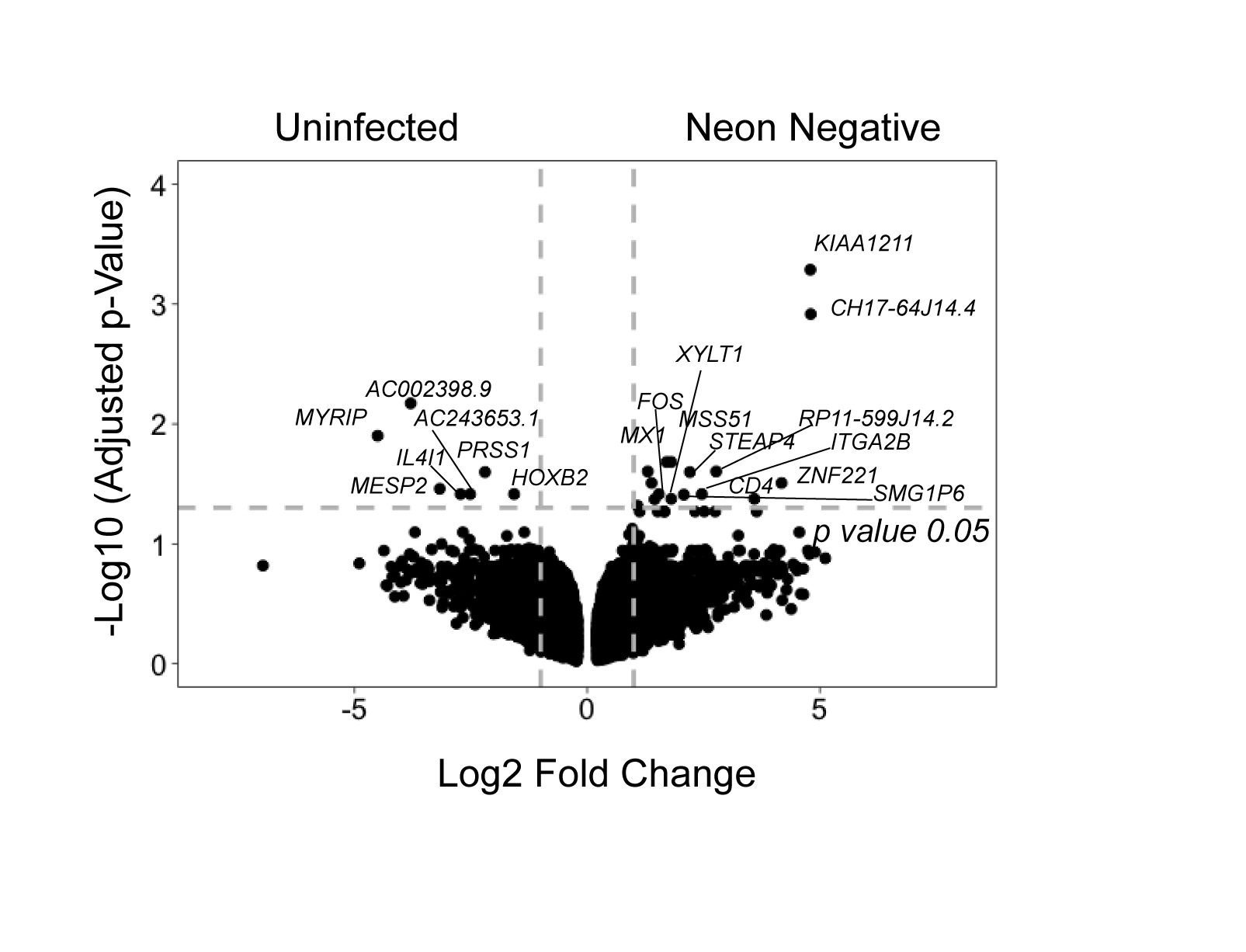

Supplement: S5 Fig — Volcano plot of RNA-seq of Neon negative bystander cells (see Fig 3H) were compared to uninfected controls. Only a relatively small number of differentially expressed genes was observed and no enrichment of an IFN signature was noted in either dataset (see S5 Table for GSEA results). (TIF) [file ppat.1010003.s005.tif]

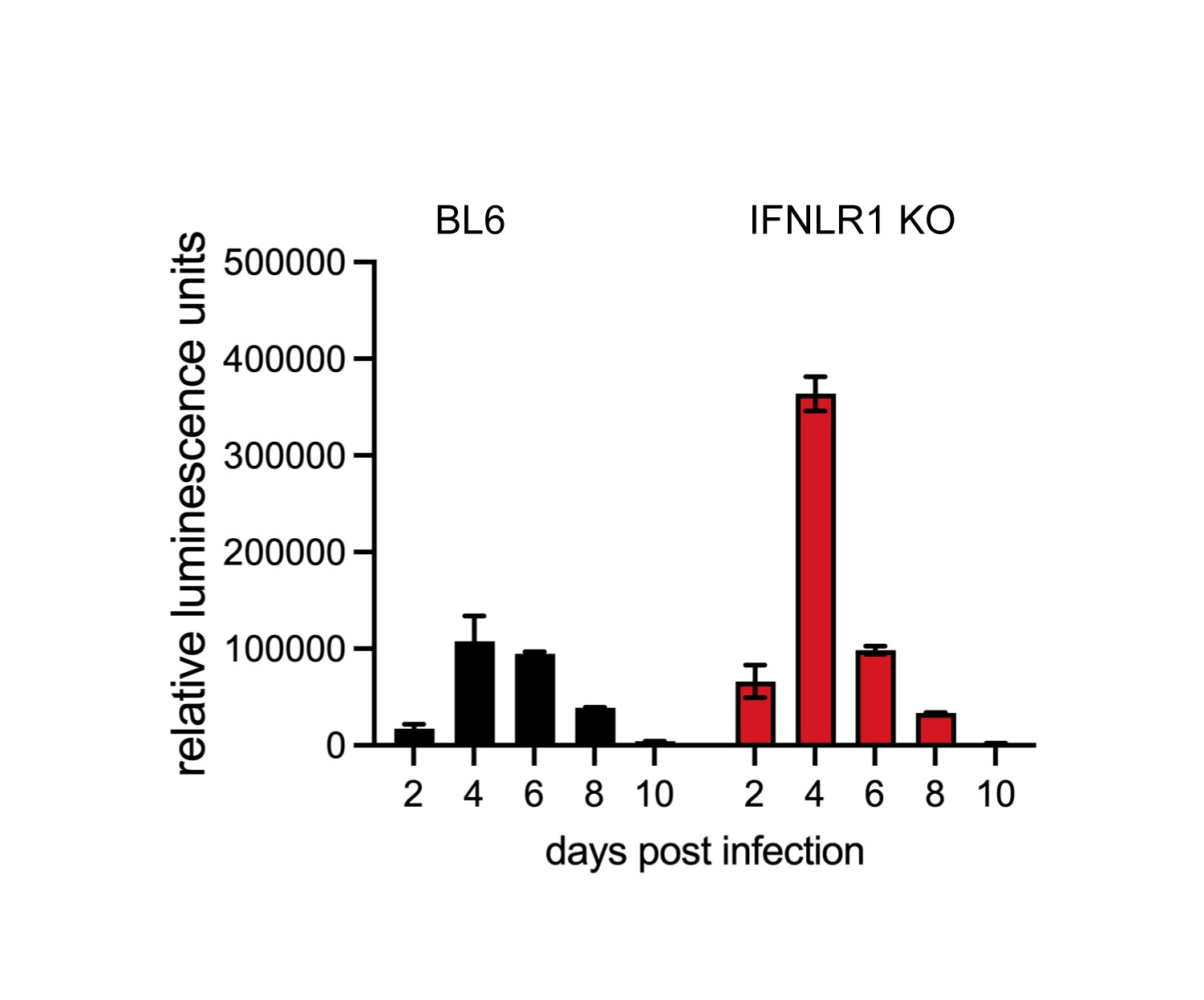

Supplement: S6 Fig — 4-week-old mice were co-housed for 3 weeks prior to infection. Fecal luminescence measured every two days in C57/BL6 wild type mice and mice lacking the type III interferon receptor Ifnlr1-/- following infection with 50,000 C. parvum. Aggregate of 2 biological replicates is shown. An average 2-fold increase was observed across 2 biological replicates. 4 mice per group. (TIF) [file ppat.1010003.s006.tif]

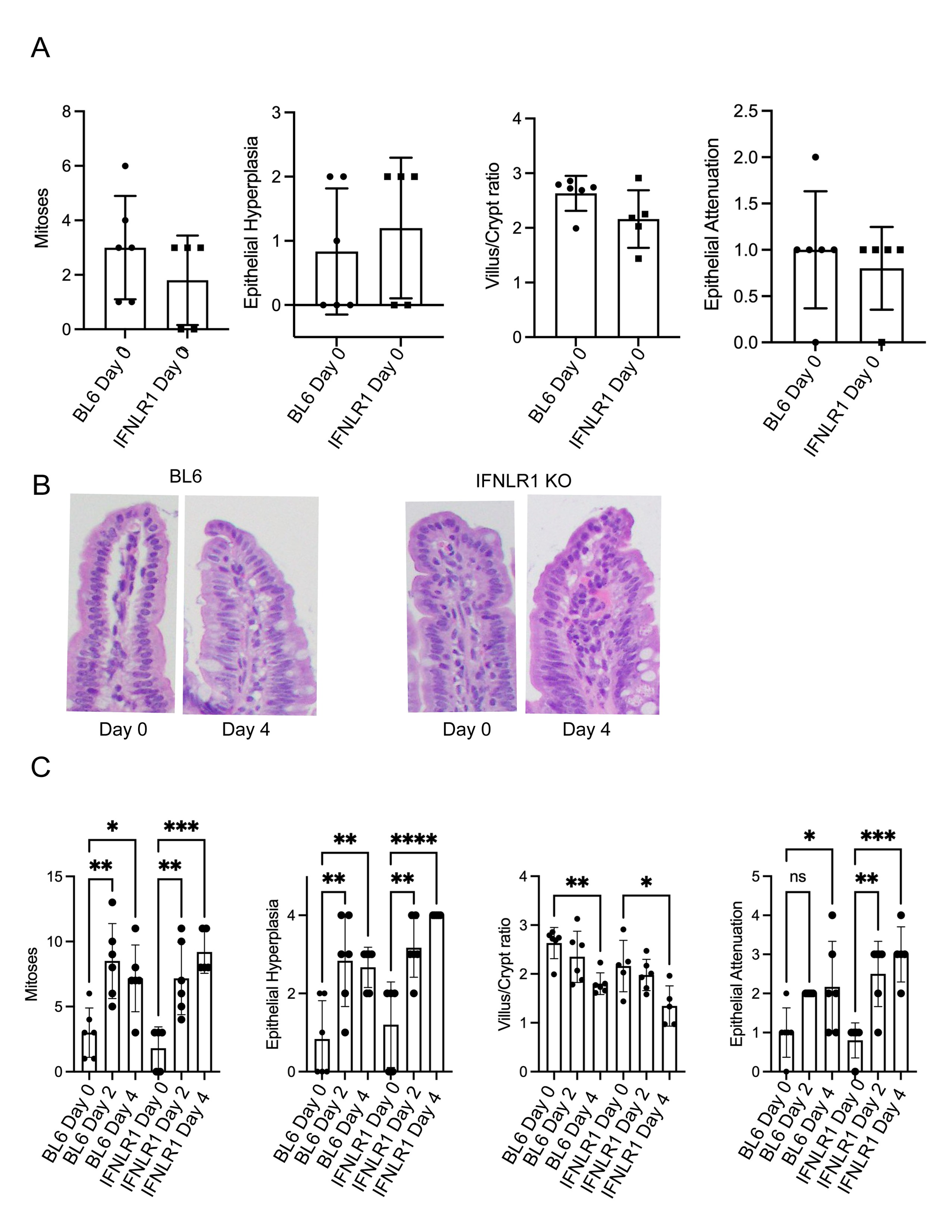

Supplement: S7 Fig — (A) Histology scoring from uninfected BL6 wild type mice and mice lacking the type III interferon receptor Ifnlr1-/-. No differences were observed. (B) Hematoxylin and eosin stained sections of the distal small intestine of BL6 and IFNLR1 KO mice prior to and 4 days post infection. (C) Histology scoring from BL6 wild type mice and mice lacking the type III interferon receptor Ifnlr1-/- infected with 50,000 C. parvum and uninfected controls. One-way ANOVA with Šídák’s multiple comparisons test * p <0.05 ** p <0.01 *** p <0.001 **** p <0.0001. Differences between uninfected Ifnlr1-/- mice compared to infection tended to be more statistically significant than those observed between uninfected and infected BL6 mice. (TIF) [file ppat.1010003.s007.tif]

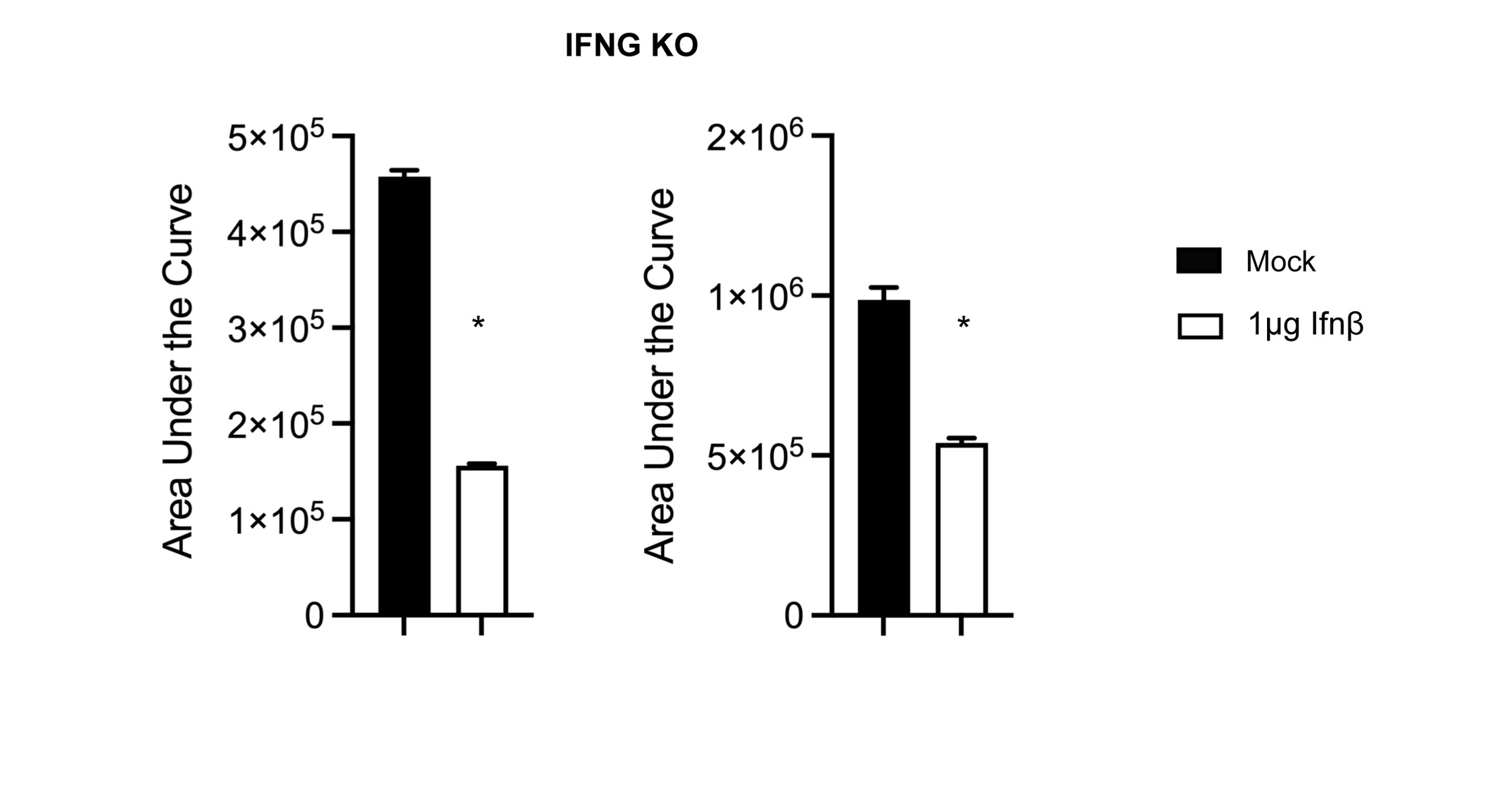

Supplement: S8 Fig — Ifng-/- mice were injected i.p. with 1μg Ifnβ daily on days 0–3 of infection. Mice were infected with 20,000 C. parvum oocysts. The total area under the curve of fecal luminescence for the 3-day infection is shown. Two biological replicates are shown resulting in a decrease of 2.9 and 1.8-fold. Standard t-test of area under the curve across two biological replicates * p <0.05. (TIF) [file ppat.1010003.s008.tif]

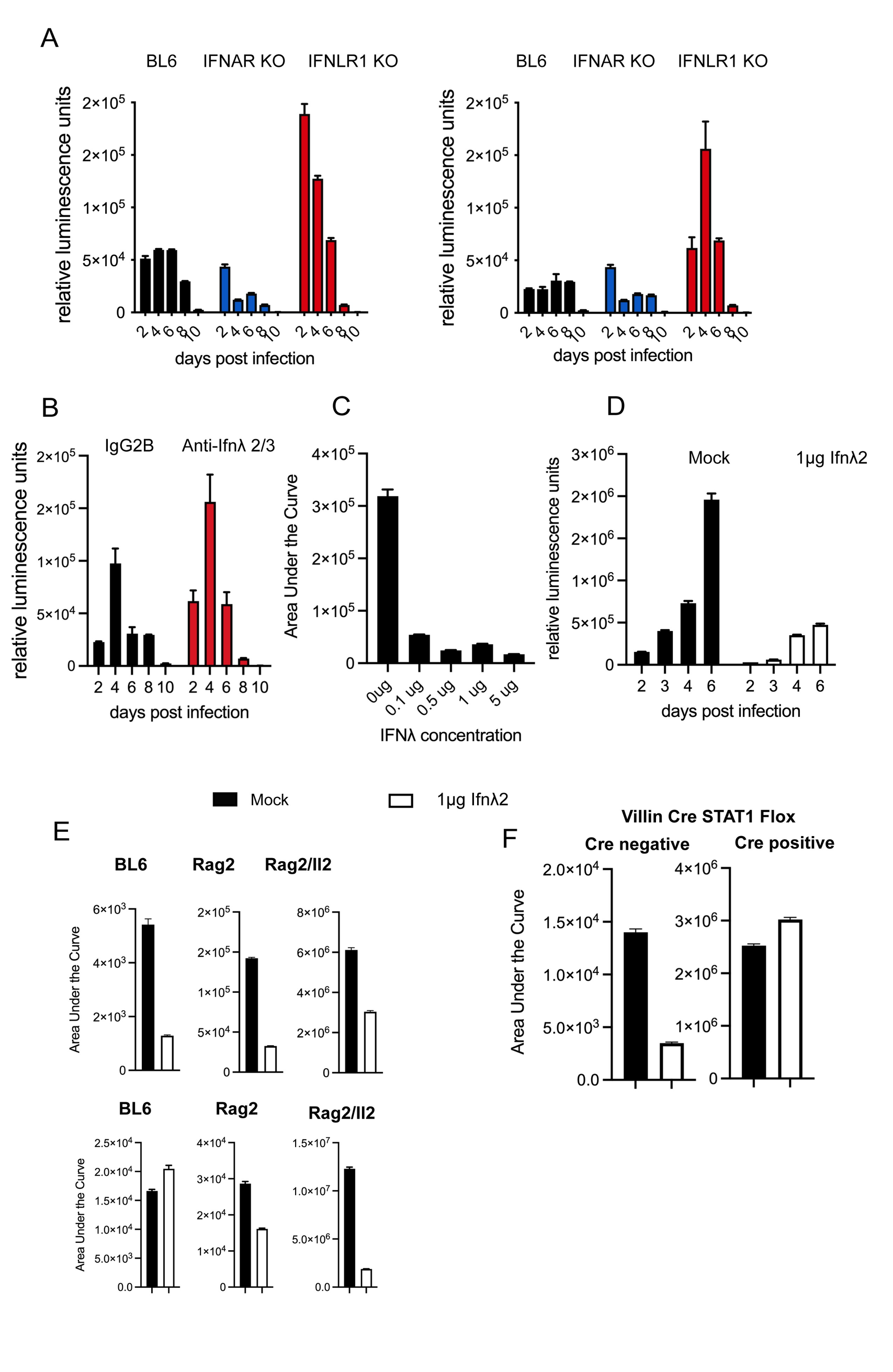

Supplement: S9 Fig — Fecal luminescence was measured every two days following infection with 50,000 C. parvum. (A) BL6 wild type mice, Ifnar1-/-, and Ifnlr1-/-4 mice per group, 2 additional biological replicates shown. (B) C57/BL6 mice were treated with anti-Ifnλ2/3 antibody or an isotype control daily via intraperitoneal (i.p.) injection and infected. One additional biological replicate shown. (C) Ifng-/- mice were injected i.p. with indicated doses of Ifnλ2 daily for days 0–3 of infection. Mice were infected with 20,000 C. parvum oocysts. The total area under the curve of fecal luminescence for the 3-day infection is shown. 2 mice per dose. One additional biological replicate shown. (D) Ifng-/- mice were injected i.p. with 1μg of Ifnλ2 beginning at day 0 and each day for the duration of the infection. Mice were infected with 20,000 C. parvum oocysts. One additional biological replicate shown. (E) Wild type mice (B6), mice lacking T cells (Rag2-/-), and mice lacking NK cells, ILCs, and T cells (Rag2/Il2rg-/-) were treated with 1μg of Ifnλ2 daily for the days 0–3 of infection. The total area under the curve of fecal luminescence for the 3-day infection is shown. Two additional biological replicates shown. (F) Villin Cre STAT1 flox mice or littermate Cre negative controls were treated with 1μg of Ifnλ2 daily for the days 0–3 of infection. The total area under the curve of fecal luminescence for the 3-day infection is shown. One additional biological replicate shown. (TIF) [file ppat.1010003.s009.tif]

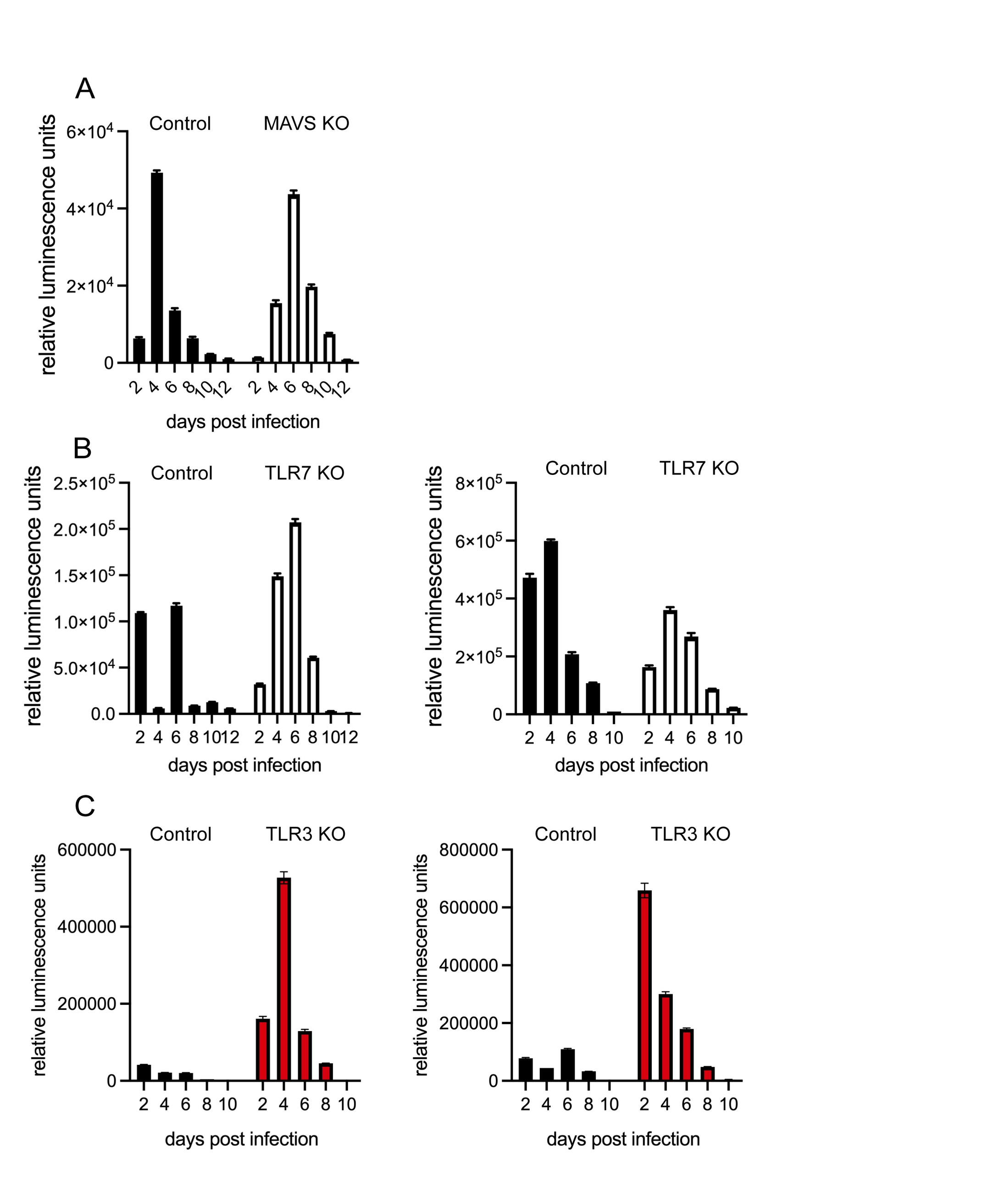

Supplement: S10 Fig — Fecal luminescence measured every two days following infection with 50,000 C. parvum. (A) Infection of wild type control mice (B6129) compared to mice lacking MAVS. One additional biological replicate is shown. (A) Infection of wild type control mice (C57B6N/J) compared to mice lacking TLR7. Two additional biological replicates shown. (C) Infection of wild type control mice (B6129) compared to mice lacking TLR3. Fecal luminescence measured every 2 days. Two additional biological replicates shown. (TIF) [file ppat.1010003.s010.tif]

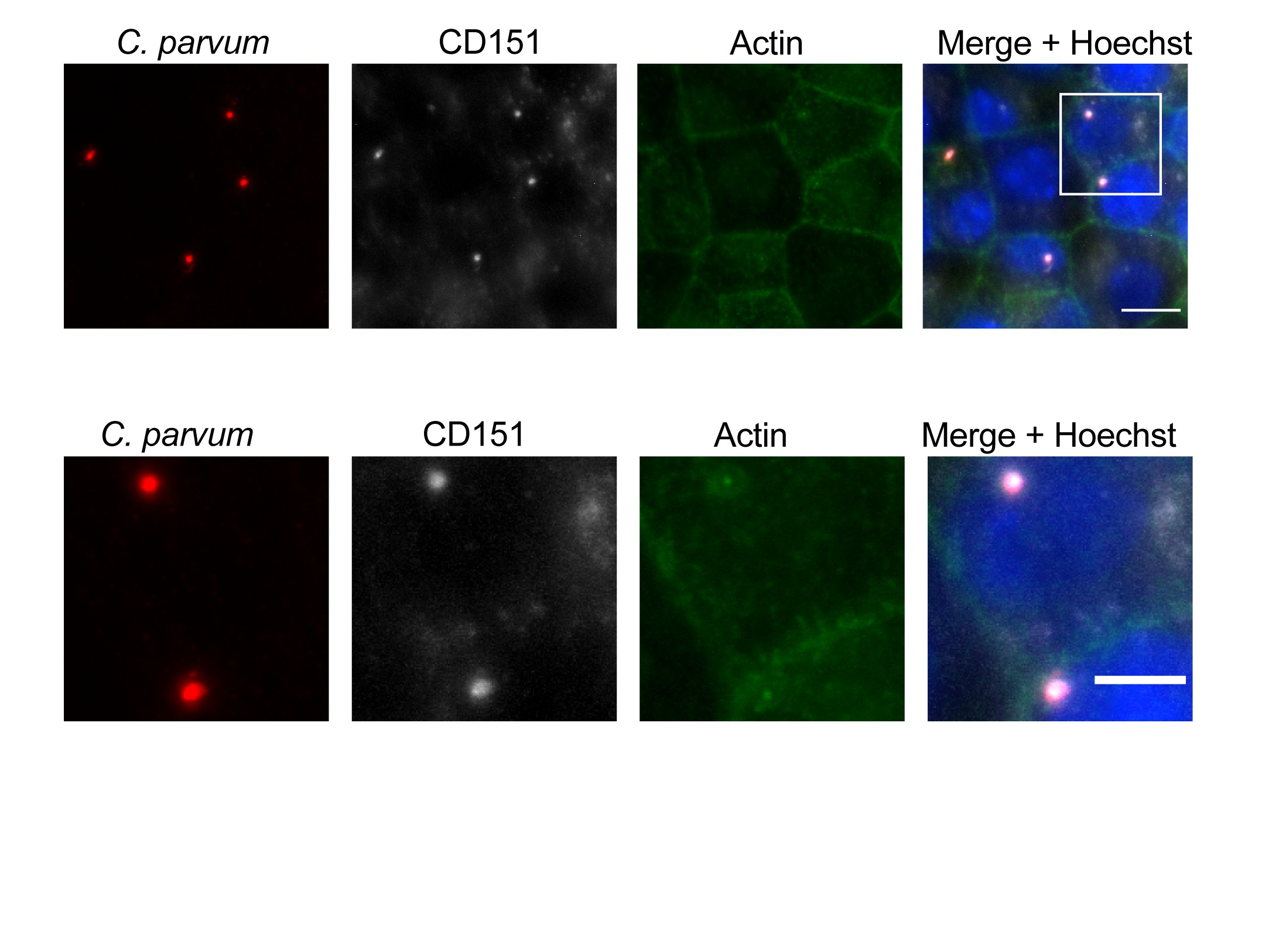

Supplement: S11 Fig — Immunofluorescence of HCT-8 infected with C. parvum at 1 hour post infection. C. parvum (red), CD151 (gray), actin (green) Hoechst label nuclei. Scale bar 10μm in top panel, scale bar 5μm in bottom panel. (TIF) [file ppat.1010003.s011.tif]
