# Supplementary material for: A genetic screen identifies a protective type III interferon response to Cryptosporidium that requires TLR3 dependent recognition
Source: PLoS Pathog. 2022 May 18;18(5):e1010003. doi: 10.1371/journal.ppat.1010003 (PMC9154123; doi:10.1371/journal.ppat.1010003)
Supplement: Attachment — Submitted filename: Rebuttal_PLoS_Pathogens_final.docx [file ppat.1010003.s017.docx]

**Response to Reviewers**

Reviewer #1: Major points:
(1) As mentioned above, there is a significant lack of connection between the CRISPR screen and IFN-λ inhibiting Crytosporidium infection in mice. For instance, does knocking out STAT1 (#1 hit) prevent Crytosporidium induced cell death in HCT-8 cells? How does that reconcile with increased Crytosporidium infection in vivo (Fig. 4H)? This needs to be discussed in the paper.

*We followed the reviewer’s suggestion, and targeted STAT1 and STAT2 for knockdown in HCT-8 cells using RNAi and measured cell death upon infection with Cryptosporidium. We found that in the absence of infection, there was no difference in cell viability between the scrambled siRNA control and the knockdown of STAT1 or knockdown of STAT2. However, following a 48hr infection with Cryptosporidium, host cell viability is significantly greater in cells targeted for STAT1 and STAT2 knockdown. These new data are now shown in supplementary figure S2 Fig.*

*As requested, we discussed this issue. Interferons can be potent inducers of host cell death. While this cell death is deleterious in vitro, in the mouse, killing of infected host cells is likely to prevent completion of the intracellular development of the parasite, which replicates by schizogony. We propose that a loss of type III IFN induced cell death may lead to the increased parasite burden seen in Ifnlr1^-/-^ mice. We have expanded the discussion to make this clearer.*

(2) I have several problems with Fig. 4. Panel (A): IFN-λ is generally rapidly induced post infection. 12 hr or at least day 1 data would be revealing (higher fold induction than day 2). Panel (B): huge error data. More replicates needed.

*We followed the reviewers request and conducted additional experiments to increase the replicates for both Fig4A and Fig4B, there are now between 8-10 mice per group for each of these panels.*

Panel (C): several virus infection studies have shown a redundant role of type I IFN and IFN-λ (Crotta S et al., PLOS Pathog, 2013; Lin JD et al., PLOS Pathog, 2016). The increase of Crytosporidium in IL28RA knockout mice was far less than that in the STAT1 floxed, Villin-Cre mice (Fig. 4H). The authors need to test Crytosporidium infection in Ifnar1, Ifnlr1 double knockout mice to show that IFN-λ alone controls infection.

*The reviewer asked us to test whether there was a difference between Ifnlr1 and Ifnar/Ifnlr1 double KO mice. We found that double KO and Ifnlr1 KO resulted in indistinguishable parasite burden, suggesting that type III IFN is driving the increased parasite shedding measured in these mice. We no longer have access to these mice as our colleague, Dr. Carolina Lopez, moved to Washington University in St. Louis. We have thus not repeated the experiment and will not include this in the manuscript but include the data here for the reviewer.*

IFNLR1/IFNAR1 KO

BL6

IFNLR1 KO

Panel (D and F): anti-IFN-β and recombinant IFN-β need to be included as controls to show specificity.

*Following the reviewer suggestion, we treated Ifng^-/-^ mice with recombinant IFN-β. Across two biological replicates, we observed a reduction of infection of 2-3-fold compared to mock treated infected mice. This data is now shown as supplementary figure S8 Fig. and described in the results section. IFNλ treatment led to a 7-fold decrease. This is reminiscent of findings by Pott et al 2011, where rotavirus infections could be reduced upon treatment with both type I and type III IFN, with type III IFN being more effective. We clarify this point in our discussion and we add this reference.*

(3) The protective role of IFN-λ in Crytosporidium infection has been reported. The cell types responsible for IFN-λ production and the upstream sensors are potentially novel and unfortunately not examined in depth. What cells (intestinal epithelial cells, intraepithelial lymphocytes, lamina propria hematopoietic cells) are the major producer of IFN-λ in response to Crytosporidium infection? The authors showed a role of TLR3 in IFN-λ expression. However, in what compartment? Do TLR3 floxed, Villin-Cre mice show the same phenotype as full TLR3 KO mice?

*Our in vitro experiments indicate that Cryptosporidium infection of IECs is sufficient to induce type III IFN production (Fig 3A, Fig3D). Further, sorting infected cells from bystanders shows IFNλ to be preferentially induced in the infected cell, not bystander (Fig 3H). These data support IECs as the major producers of Ifnλ upon infection. In contrast, neither B nor T cells have been shown to produce Ifnλ in mice (Broggi et al. 2017, Galani et al. 2017), similarly dendritic cells in mice have not been shown to contribute to IFNλ production, and neutrophils and NK cells respond to Ifnλ but not to produce it (Broggi et al. 2017, Gimeno Brias et al. 2018). The data presented here supports the conclusion that IECs are the primary producers in this infection, however, we now acknowledge that we do not formally exclude the possibility of other sources.*

Reviewer #2: 1. What happens when you perform 3 rounds of 90% killing dose on non library-transduced cells? Is this dose sufficient to kill all host cells, or do you select for spontaneously resistant cells? If yes, it might be interesting to test expression of interferon stimulated genes in those cells. Please comment on this.

*Thank you for this question. We conducted this experiment and note no change. This important piece of data is now shown in supplementary figure S1 Fig.*

2. Figure 1A: at the dose where you obtain 90% killing, are all your cells infected with the parasite? How can you exclude the influence of death of bystander cells? Please show infection rate in addition to cell viability data. Did you perform technical and biological replicates (different batches of Crypto) in the infection assay? Can you make it a bit clearer what MOI you used?

*Different batches of Cryptosporidium indeed produced slightly different kill curves, we expect this was due to differences in viability and excystation rate of the parasite. For this reason, the entire screen was performed with the same batch of Cryptosporidium oocysts. The MOI at a 90% kill dose is 3 sporozoites to 1 host cell and we now provide this number. Even at 99% kill, not all host cells are infected. We cannot exclude impact on bystander cells, and we do not claim to do so.*

3. Genome wide screening data is shown from clone K (Figure 1D) and clones K+I (Figure 1E). What about the data from clone C? Why was clone F excluded, seeing as this has the highest level of Cas9 activity? Please illustrate how well the hits from your three replicates overlap with each other.

*Experiments using Clone C resulted in poor sequence coverage, this was mentioned in the methods and is now stated in the results. The IFN signature was identified in all three analyses, with STAT1 and STAT2 being significant hits in all three replicates of the screen. Analysis of Clone C alone resulted in only 8 hits. Individual analysis of Clone I and Clone K resulted in 10 and 14 hits, respectively, with some common hits falling just above the level of significance. Analyses of Clone I and Clone K alone both identified the top signatures described in the manuscript of IFN signaling, GPI anchor biosynthesis, and GAG biosynthesis. This expanded analysis is now included in supplemental figure S3 Table.*

*Clone F was not used for the screen as that clone became contaminated.*

4. It seems to me that 3 rounds of 90% killing dose is a very stringent selection, and that it’s possible (likely?) you enriched for genes that are essential for early steps in parasite attachment and invasion and lost hits that may be essential for parasite development. I think this is worth commenting on.

*This is a good point, and we now emphasize this in the discussion.*

5. After each round of infection, you collected DNA from cells that did not die. Can you distinguish between cells that did not become infected vs cells which became infected but did not die? Some microscopy to check presence / absence of the parasite in the surviving cells would have been interesting. Do you have any material remaining with which you can estimate the level of infection in the surviving cells? I find the subtitle “Genetic screen reveals genes required for infection and host response” a little misleading as I do not think you distinguish between these aspects. I don’t understand from your data whether knock out of your 35 top hits blocks invasion of the parasite, blocks progression of parasite life cycle development, or suppresses the host response (inflammatory? cell death?) to parasite infection. Please comment.

*We think that our screen identifies genes that are important for both infection and/or host cell survival. Though we do not know what each individual hit is responsible for, we highlight the potential roles in both parasite and host processes in the discussion of individual hits.*

*We followed up on the reviewer’s suggestion with experimentation. We did not freeze cells after each selection, but we did so, following the complete selection. In response to the question, we infected those cells and note that they showed reduced infectivity, as suggested by the reviewer. They also showed a trend of decreased cell death upon infection. We now present this data in supplementary figure S3 Fig.*

6. A follow on from point 5 – I suggest that you do a more in-depth characterization of selected candidates (in particular those from the interferon pathway) and analyse in vitro at which point parasite invasion / development / egress is blocked. Monoclonal Cas9 knock outs would be great, but you can probably also obtain this info from your siRNA knock downs.

*We infected post-screen and pre-screen cells and analyzed a time course of infection. We find significant reduction in parasite burden both early and late during infection. These data are now presented in supplementary figure S3 Fig panel C. In an effort to further follow the reviewer’s suggestion, we engineered monoclonal Cas9 mutants in STAT1, STAT2 and IRF9 and these indeed result in significantly decreased parasite growth. However, in complementation experiments using overexpression of the respective cDNA clones we did not observe a reversal of this phenotype. We therefore decided not to include these experiments in the revised manuscript and instead focus on the siRNA experiments discussed in response to reviewer #1.*

7. Fig S1B/C. The cell viability in the HCT-8 transduced with scrambled siRNA is decreased upon infection, but I would have expected a much more significant decrease. Were you expecting this result? Did you infect the cells with a 90% kill dose, like in the screen? Why was viability not assessed at 72 hours post infection (particularly considering the significant upregulation of ISG transcripts at 72h).

*A technical limitation faced here is that HCT-8 cells are poorly transfectable and RNAi knock downs are modest and transient. Target mRNAs show significant rebound at 72 h and we therefore screened at 48 h. As shown, the average percent knockdown varies from 30-90% loss of transcript. We thus used a lower dose of Cryptosporidium to avoid excessive cell death. The MOI used here was 0.8 and we now state so and discuss the limitation of transformation efficiency.*

8. Figure 4: Here mouse models lacking IFN Type 1 receptor (ifnar -/-) or IFN type III receptor (Il28ra-/-) were used. Loss of type 1 receptor resulted in a reduction in oocyst shedding, while loss of type III interferon signaling resulted in an increase in parasite shedding. Data from the knock out mice was supported nicely with antibody blocking experiments. Together this showed that interferon type III signaling provides a protective response in early infection. It would help to tie this observation back to the data from the genome wide screen here, where you showed that losing interferon-signaling related genes blocked cell death in vitro. I must admit I got a bit lost here and would appreciate some more clarity in the discussion.

*Interferons can be potent inducers of cell death. While this cell death is deleterious in vitro, in the mouse, killing of infected host cells is likely to be protective as the intestine is constantly renewed with new epithelial cells. We now discuss this in greater detail.*

9. Figure 4: Parasite load was increased in mice lacking the type 1 interferon receptor (Ifnar-/- mice). This is a surprising observation. What is your explanation for this?

*The parasite load was decreased in Ifnar1^-/-^ mice, that was surprising, and we state so explicitly in the results. Considering the literature on the decreased function of regulatory T cells (Treg) in Ifnar^-/-^ mice this may be the result of activation of effector T cells at baseline (Metidji et al. 2015). This has been shown specifically in the gut in a model of T cell mediated colitis where effector T cells proliferated rapidly and accumulated which coincided with an impairment in Treg accumulation (Kole et al. 2013). We now introduce these thoughts and references in the discussion.*

Reviewer #3: 1. Regarding the protective role of type 3 IFNs during cryptosporidiosis: The data presented present an apparent contradiction between the initial screen, showing several genes involved in the IFNs signaling pathway as important determinants of host cell death following infection, and the subsequent experiments showing the protective role of type III IFNs during cryptosporidiosis. If deleting these IFN signaling genes (i.e. STAT1/2, TYK2, IFNAR, IL10RB, IFNLR1, JAK1 and IRF9) strongly increased survival of host cells in vitro, how do the authors explain the protective role of this same pathway in vivo? In the discussion of this paper, they suggest that these observations might be the result of IFNs (type I and III in this instance) inducing cell death following their upregulation by infection. Unfortunately no support is provided for this hypothesis. It would be important to test whether administration of type 3 IFNs at the level seen in cells following infection are capable of inducing any cell death program – or if this outcome also requires infection.

*Thank you for this suggestion. We now provide additional datasets that show that administration of IFNλ in our HCT-8 cells indeed leads to a decrease in cell viability. This occurs even in the absence of infection and infection appears to be a stronger driver of cell death than IFNλ alone, likely because infection leads to production of IFNλ as well as cell lysis by parasite egress. These data are now presented in supplementary figure S4 Fig panel C.*

2. Regarding the type 1 IFNs results: How do you explain the growth facilitating effect of type I IFNs observed in vivo (lower burden in Ifnar mice compared to WT in Fig 4C) – which could be consistent with the results of the in vitro screening? It seems that the authors focused their efforts on type III IFNs since the qPCR assessment of gene expression after infection showed almost no increase in expression of IFNB (Fig 2E). However, of the genes found significantly enriched in the screen, only the IFNLR subunits are not part of the type 1 IFNs pathway, and one type 1 IFNs receptor subunit was included. It would be beneficial to examine KO cells lines to decipher the role of each one of these cytokines during infection in vitro. Does loss of production of either type I or type III IFN, or their respective signaling receptors, protect cells against death due to infection?

*Loss of interferon signaling does reduce host cell death in vitro due to infection, please see our response to reviewer #1, question 1. We also ask that you see our response to reviewer #2, question 9 on this point above.*

3. Regarding the role of TLR3 in inducing the expression of type 3 IFNs following infection with Cp: In the initial testing of PRRs (Fig 5A), dsRNA did not induce the expression of IFNλ. In contrast, the authors propose that dsRNA – possibly originating from parasite viruses – is recognized by TLR3 to induce an immune response. However, it is hard to understand why these two different forms of dsRNA would have such different outcomes. Do you have any insight about how intraparasitic virus-derived dsRNA could end up exposed to endosomal TRL3?

*Please note that dsRNA DOES induce, Poly (I:C) treatment with or without lipofection induced IFNλ production. It appears that our description in Fig. 5 was confusing, and we now make this clearer.*

Furthermore, you discuss the role of type 1 IFNs expression induced downstream of TLR3 activation in leishmaniasis, leading to disease exacerbation. In contrast, type I IFN does not seem to be strongly stimulated by C. parvum, despite evidence for signaling through TLR3. Might the differences observed be due to the fact that the two types of target-cells are widely different (IECs in cryptosporidiosis vs MΦs in leishmaniasis), or be due to some parasite-induced type 1 IFNs-inhibition mechanism? In this regard, you did measure IFNβ expression in HCT-8 cells following lipofection with poly(I:C) (Fig S2) which also activates TLR3.

*This is an excellent point; indeed, the target cells are very different. IECs are highly skewed towards type III IFN production. We propose that the differences are primarily due to differences in the cell type infected. We now feature this in the discussion. At this time we do not have evidence for a Cryptosporidium specific mechanism of type I IFN inhibition.*

*We modify the discussion to now read:*

*For Leishmania, TLR3 recognition was dependent on the presence of a dsRNA virus found in certain parasite isolates. Interestingly, Cryptosporidium is also host to a dsRNA viral symbiont which could be a possible source of dsRNA recognized by TLR3 (Khramtsov et al. 1997, Khramtsov et al. 2000). In contrast to leishmaniasis where type I IFN production exacerbates disease (de Carvalho et al. 2019), the type III interferon response to C. parvum is host protective this is likely due to the different cell types, macrophages for Leishmania and intestinal epithelial cells for Cryptosporidium. There have also been reports of Cryptosporidium derived RNAs to be trafficked into the host cell nucleus during infection providing an additional potential trigger for TLR3 (Wang et al. 2016). The route and mechanism by which such parasite derived RNA molecules would traffic into the host cell is unknown.*

Reviewer #4: Major comments:
1. The conclusion on line 263 that “…induction of the interferon pathway is exclusive to infected Neon positive cells” is not well supported by the data and the authors should consider revising. As I understand the experiment is comparing infected to bystander rather than to uninfected, so it could be that the bystanders are responding vs uninfected, but with reduced magnitude compared to infected. Additionally, it would be interesting to know whether any IFN or other gene signatures are present among the large number (466) of downregulated genes in infected vs uninfected.

*This is an excellent point. We now feature an additional comparison of our Neon negative bystander cells to uninfected; these data are now shown in S5 Fig and support the statement that induction of the interferon pathway is exclusive to infected cells. The Neon negative cells are not enriched for interferon signaling genes compared to a true uninfected control. The GSEA results for the uninfected to bystander comparison are now included in S5 Table.*

*While we focus our comments on the upregulated genes, all differentially expressed genes and signatures for the 10hr dataset are now presented in the S5 Table. The loss of the downregulated genes was an oversight and has been corrected. Only 4 pathways were significantly enriched in the Neon negative population. Signatures found in the downregulated genes of the 10hr dataset appear to be linked to transport of small molecules, inorganic cations and amino acids.*

2. Infection data in figures 4 and 5 need statistical analyses to back up conclusions regarding fold-change of AUC stated in the text. Conclusions drawn in text appear to be well-supported by data, but clarity in results of statistical tests are required.

*We show representative infection curves throughout, and each individual biological replicate can now be found in the supplement. Statistical tests were performed on the fold change of the area under the curve using all replicates. For example, in Fig 4C we normalize BL6 infections to 1 and report a decrease in Ifnar1 infections by 3, 2.8, and 2.9-fold and an increase in Ifnlr1 infections by 2.7, 2.3, and 2.7-fold. A one-way ANOVA was then performed on these summary data and the results of the statistical test reported in the main figure [please see graphical representation below]. We make this clearer now in the materials and methods section.*

3. Mouse phenotypes (especially gut phenotypes) can be impacted by microbiota or other environmental factors and controlled by use of littermates or cohousing. From methods, it seems that littermate mice or cohousing were not part of the experimental design (with perhaps exception of the Vil-cre/Stat1flox experiment?). Authors should clarify whether littermates were used or if co-housing of knockout with WT was employed for any of the experiments.

*It is difficult for us to cohouse mice during these experiments because Cryptosporidium is highly infective and transmitted by the fecal-oral route. We note that we have previously conducted cohousing experiments in studies of the role of the NLRP6 inflammasome (Sateriale et al. 2021). We did not find a significant impact on parasite burden upon cohousing. We conducted cohousing experiments with IFNLR1^-/-^ mice and C57/BL6 mice for 3 weeks prior to infection. Again, cohousing did not impact the results of infection, we now show this in S6 Fig. As suggested, we now clarify explicitly in the Material and Methods that mice were not cohoused, and we directly point readers to the supplementary figure.*

**Part III – Minor Issues: Editorial and Data Presentation Modifications**
Please use this section for editorial suggestions as well as relatively minor modifications of existing data that would enhance clarity.

Reviewer #1: Minor points:
(1) Author Summary, lines 52-53, the meaning of IFN-λ “induction likely preceding IFN-γ” is unclear, as a careful comparison of temporal dynamics was not carried out in the study.

*We removed the reference to IFNγ.*

(2) A standard CRISPR/Cas9 knockout screen was carried out in the study. Fig. 1A-D is not necessary and does not add to the paper.

*Other reviewers asked for additional information on how the screen was performed suggesting this information may help the overall understanding of the study for readers not as familiar with this as the reviewer.*

(3) Top up-regulated and downregulated genes need to be highlighted in Fig. 2A and 3H. Otherwise, the graphs are not informative.

*Select gene symbols are now labeled on the volcano plots.*

(4) Fig. 2E and F, IFN-β expression was not induced by Crytosporidium infection. What about the different IFN-α isoforms? Can the authors comment on why IFNAR2 was identified in the screen if HCT-8 cells do not produce type I IFNs?

*We did not examine the expression of the different IFNα isoforms. IFNβ has the ability to be produced by virtually all cell types which is why we examined expression of this type I IFN specifically.*

(5) ISGs upregulated in RNA-seq data in Fig. 3H need to be validated by QPCR.

*ISGs such as IFNL1 and OASL were validated by qPCR (Fig 2C and E).*

(6) Genetic background of mice and knockout mice used in Fig. 5 should be labeled in the graph.

*This background information was added.*

(7) Fig. S3, immunohistology data of intestinal sections should be provided here.

*Representative images are now included.*

(8) The complete analysis of CRISPR screening data needs to be provided in S3 Table.

*The complete analysis is now provided.*

(9) Proper statistical analyses are needed for all column graphs throughout the manuscript.

*Statistical analyses for bar graphs are now included throughout.*

Regarding RNA-seq:
(1) What percent of reads aligned to the human genome? What percent to the cryptosporidium genome? The discrepancy in IFNL transcripts quantified with RNA-Seq and by qPCR may be explained by this.

*At least 85% of reads aligned to the human genome in all samples and only 3% or less of our reads mapped to the Cryptosporidium genome and we describe this in detail now in the materials and methods.*

(2) What type of transcript was considered a differentially expressed gene: protein-coding, noncoding, small RNAs, etc?

*We did not exclude non protein-coding genes in our analysis, any transcript with a corresponding gene ID in the Ensembl 86 version of the human genome was included in the initial read mapping.*

(3) The color-coding of the volcano plots in Figure 2A and Figure 3H are confusing: typically, a non-black color is given to all differentially expressed genes in a volcano plot while those that are not DEGs are black. It isn’t clear, unless reading the legend, that only genes enriching to a particular ontology term are colored red.

*We have removed the color coding from both volcano plots and label select top genes instead.*

(4) The enrichments and genes for the 48-hour results are provided in the supplement with the text focusing on upregulated gene enrichment. It would be interesting to comment on what is downregulated. Are genes related to GAG and GPI biosynthesis included in any of these enrichments?

*Enrichment in the uninfected population focused largely on pathways related to metabolism and translation. This is possibly due to the fact that uninfected cells are still rapidly growing and not being killed by the parasite.*

(5) Please be consistent when referring to transcriptomics: the manuscript alternates between “RNAseq” and RNA-Seq.”

*Corrected.*

Reviewer #2: Point out naming convention for the interferon type III receptor (Il28ra encodes for IFNLR1) to save confusion to non-interferon specialists.

*We elected to use the updated name IFNLR1 throughout for clarity.*

Line 297: briefly comment on the phenotype of the ifng-/- mice (i.e. no interferon response at all?)

*Ifng^-/-^ mice experience a 2-log fold higher parasite burden compared to wild type mice. This is now stated in the results.*

Line 305: in contrast to mock injected mice….. no mortality. Can you show this data? How many mice died and on which day of infection?

*We now state in the text that 100% mortality was observed in the mock injected group at day 14 post infection.*

Line 361: Typo “Papillomavirus”

*Corrected.*

Line 431: Typo “TL3”

*Corrected.*

Reviewer #3: * Fig 4B: the D2 time-point shows a very high SE. Is this figure aggregating the results from the 2 replicates or just from 1 experiment? In this latter case, it would mean that only 1 of 2 mice displayed a detectable IFNλ level (I suppose that the dotted line represents the limit of detection), making the present representation a bit misleading in my opinion.

*Additional replicates have been added to Fig4A and 4B reducing the SE shown here.*

* Fig 4G: In the text, you state (discussion): “Consistent with this idea treatment of Rag2 -/- Il2rg -/- was repeatedly less effective than that of Rag2 -/- alone (Fig 4G), suggesting a potential role for IFNλ in promoting IFNγ production in NK/ILCs”. This is contradicted in the result section: “BL6, Rag2 -/- , and Rag2 -/- Il2rg -/- mice were infected and treated with 1μg of Ifnλ2. This resulted in comparable reduction of parasite shedding (BL6: 2.4-fold, Rag2 -/- : 4.4-fold, Rag2 -/- Il2rg -/- : 2-fold, Fig 4G), again suggesting that the benefit of IFNλ treatment does not require immune cells, but largely rests on an enterocyte intrinsic response”. Furthermore, the choice of representative figures for these 3 replicates is a bit misleading, presently showing BL6 treatment as highly effective, whereas, per the results presented in the text, it is not much more effective than Rag2 -/- Il2rg -/- treatment.

*We recognize the confusion on this point and elected to remove this statement from the discussion for clarity.*

* Fig 4 legends: Many of these examples report showing a “representative example of several biological replicates”. The sample sizes are quite small here. It would be preferable to show aggregate data with combined statistical analysis for all biological replicates – or alternatively show the other biological replicates in the supplement. If you chose this option, in the text, please specify if the same statistical findings were seen in all replicates or only some.

*All additional replicates can now be found in the supplement. We also clarify that each replicate includes 3-4 mice per group.*

* Fig 5E: the results section states: “We found the production of IL-18, an enterocyte derived cytokine induced by Cryptosporidium infection, [46, 47] to be intact in the absence of TLR3 (Fig 5E)” but, in fact, these results show the IL-18 expression to be increased in TLR3 KO mice by more than 2 fold in a consistent way. Is it possible that the increased parasite burden due to TLR3 ablation is responsible for this increased expression of IL-18? Did you check if this increase is correlated with an increase in IFNγ production in these mice? If so, this result might contradict the claim that there is “a potential role for IFNλ in promoting IFNγ production in NK/ILCs”?

*Excellent point, we removed this sentence in the discussion in response to another reviewer comment.*

Reviewer #4: Minor comments:
4. Fig 3H. In this representation the ISGs (red) are identified by Interferome DB rather than the curated REACTOME gene set used in earlier figure 1A-B. It would be nice to keep the signature used for ISGs consistent between these gene expression studies in different figures.

*We have removed the color coding on both volcano plots as one reviewer stated that it was confusing.*

5. Lines 278-280. The type I interferon receptor is a heterodimer of IFNAR1 and IFNAR2, but text implies that there is only one IFNAR gene. Authors should clarify this point in the text, and be clearer about which knockout was used (Ifnar1 as per methods).

*We have clarified these points in the text.*

6. Authors may want to use the updated ncbi gene name Ifnlr1 (rather than Il28ra) when referring to the knockout mouse because this is more clearly matching the IFNL cytokine nomenclature.
7. Authors should indicate the specific strain of Ifnlr1 knockout in the methods. If it was obtained from BMS, I presume it the Ifnlr1tm1Palu line originally generated by Ank et al. 2008 (10.4049/jimmunol.180.4.2474).

*The reference to this paper is now included.*

8. Lines 1109-1110. This panel description is confusing to read. Maybe add parentheses around mouse knockout names?

*We have added parentheses around the knockout names to make the figure legend clearer.*

9. Labels to identify bars in panels 4G and 4H are absent. I presume that open bars are IFNL treated as in panel 4F, but this should be clarified in the legend.

*We have added these labels in the figure legend.*

10. Line 297. The authors should include a rationale for switching to use ifng-/- mice for the IFNL therapeutic experiments. Was IFNL treatment ineffective in WT?

*IFNL treatment was effective in WT mice. We chose to use Ifng ^-/-^ mice to provide a greater dynamic range to measure differences in infection following IFN treatment. This rationale is now included in the text.*

11. Line 304-305. The authors indicate a difference in mortality, but should also report numbers if they wish to draw a conclusion about about mortality difference between groups.

*We now state in the text that 100% mortality was observed in the mock injected group at day 14 post infection.*

References:

Broggi, A., Y. Tan, F. Granucci and I. Zanoni (2017). "IFN-λ suppresses intestinal inflammation by non-translational regulation of neutrophil function." Nature Immunology **18**(10): 1084-1093.

de Carvalho, R. V. H., D. S. Lima-Junior, M. V. G. da Silva, M. Dilucca, T. S. Rodrigues, C. V. Horta, A. L. N. Silva, P. F. da Silva, F. G. Frantz, L. B. Lorenzon, M. M. Souza, F. Almeida, L. M. Cantanhêde, R. d. G. M. Ferreira, A. K. Cruz and D. S. Zamboni (2019). "Leishmania RNA virus exacerbates Leishmaniasis by subverting innate immunity via TLR3-mediated NLRP3 inflammasome inhibition." Nature Communications **10**(1): 5273.

Galani, I. E., V. Triantafyllia, E.-E. Eleminiadou, O. Koltsida, A. Stavropoulos, M. Manioudaki, D. Thanos, S. E. Doyle, S. V. Kotenko, K. Thanopoulou and E. Andreakos (2017). "Interferon-λ Mediates Non-redundant Front-Line Antiviral Protection against Influenza Virus Infection without Compromising Host Fitness." Immunity **46**(5): 875-890.e876.

Gimeno Brias, S., M. Marsden, J. Forbester, M. Clement, C. Brandt, K. Harcourt, L. Kane, L. Chapman, S. Clare and I. R. Humphreys (2018). "Interferon lambda is required for interferon gamma-expressing NK cell responses but does not afford antiviral protection during acute and persistent murine cytomegalovirus infection." PLOS ONE **13**(5): e0197596.

Khramtsov, N. V., P. A. Chung, C. C. Dykstra, J. K. Griffiths, U. M. Morgan, M. J. Arrowood and S. J. Upton (2000). "PRESENCE OF DOUBLE-STRANDED RNAs IN HUMAN AND CALF ISOLATES OF CRYPTOSPORIDIUM PARVUM." Journal of Parasitology **86**(2): 275-282.

Khramtsov, N. V., K. M. Woods, M. V. Nesterenko, C. C. Dykstra and S. J. Upton (1997). "Virus-like, double-stranded RNAs in the parasitic protozoan Cryptosporidium parvum." Molecular Microbiology **26**(2): 289-300.

Kole, A., J. He, A. Rivollier, D. D. Silveira, K. Kitamura, K. J. Maloy and B. L. Kelsall (2013). "Type I IFNs Regulate Effector and Regulatory T Cell Accumulation and Anti-Inflammatory Cytokine Production during T Cell–Mediated Colitis." The Journal of Immunology **191**(5): 2771.

Metidji, A., S. A. Rieder, D. D. Glass, I. Cremer, G. A. Punkosdy and E. M. Shevach (2015). "IFN-α/β Receptor Signaling Promotes Regulatory T Cell Development and Function under Stress Conditions." The Journal of Immunology **194**(9): 4265.

Sateriale, A., J. A. Gullicksrud, J. B. Engiles, B. I. McLeod, E. M. Kugler, J. Henao-Mejia, T. Zhou, A. M. Ring, I. E. Brodsky, C. A. Hunter and B. Striepen (2021). "The intestinal parasite Cryptosporidium is controlled by an enterocyte intrinsic inflammasome that depends on NLRP6." Proceedings of the National Academy of Sciences **118**(2): e2007807118.

Wang, Y., A.-Y. Gong, S. Ma, X. Chen, Y. Li, C.-J. Su, D. Norall, J. Chen, J. K. Strauss-Soukup and X.-M. Chen (2016). "Delivery of Parasite RNA Transcripts Into Infected Epithelial Cells During Cryptosporidium Infection and Its Potential Impact on Host Gene Transcription." The Journal of Infectious Diseases **215**(4): 636-643.
